# Application of experimental design to generate relevant information and representative calibration data

**DOI:** 10.1155/S1463924697000278

**Published:** 1997

**Authors:** Suzanne Schönkopf, Dominique Guyot

**Affiliations:** 1 Camo ASA PO Box 1405 Oslo N-0115 Norway; 2 Camo ASA Olav Tryggvasonsgt Trondheim 24 N-7011 Norway

## Abstract

The basic requirement for a good calibration is representative data. This paper outlines techniques for selecting samples from an existing population. The concept of factorial designs is explained, and three ways of applying experimental design to generate representative data are described. These are: to vary the experimental conditions; focus on some of the parameters of interest directly (reference values); to vary the underlying conditions which generate consistent variations in the spectra,for example production factors. Finally the paper gives an example of the use of the concept of experimental design to pick out samples from a population.


Journal of Automatic Chemistry, Vol. 19, No. 6 (November-December 1997) pp. 225-233

### Application of experimental design to generate relevant information and representative calibration data

**Suzanne Schönkopf**
*Camo ASA, PO Box 1405, N-0115, Oslo, Norway*

**and Dominique Guyot**
*Camo ASA, Olav Tryggvasonsgt. 24 N-7011 Trondheim, Norway*

*The basic requirement for a good calibration is representative data. This paper outlines techniques for selecting samples from an existing population. The concept of factorial designs is explained, and three ways of applying experimental design to generate representative data are described. These are: to vary the experimental conditions; focus on some of the parameters of interest directly (reference values); to vary the underlying conditions which generate consistent variations in the spectra, for example production factors. Finally the paper gives an example of the use of the concept of experimental design to pick out samples from a population.*

### Representative data

One of the most important issues in calibration (for example, making a calibration model for prediction of constituents from spectroscopic data) is to use a calibration sample set that is representative for the samples to be predicted in the future. The data should span all important variations, both with regard to variability and levels. An even distribution of the samples, and a balanced data set improves the chances for a successful application. There should also be enough samples. Information equals variations, so to understand the structure of a system, or a process, data that span all important variations must be collected. This is necessary for investigating correlations in historical data, exploring causal relationships through experiment and building calibration models for prediction.

### Experimental design

Statistical experimental design [1, 2] aims at generating maximum information from a minimum of experiments. Information equals structured variation, so experimental design is used to create structured variation in experimental data.

In factorial designs, each experimental factor is varied at $N$ levels, and all combinations (or a smart subset of combinations) are tested. The simplest form for factorial design uses only two levels—each factor is varied from a low (designated $-$) to a high level (designated $+$), (table 1) with such a screening design, it is easy to find which experimental factors and interactions have a significant effect on the response (causal relationships), to investigate if the system is linear or not, and to optimize. But, since the design creates data that vary, experimental design can also be used to generate different samples, for example for calibration.

The number of possible combinations rapidly increases with increasing number of experimental factors. Therefore, there are alternative designs, for example fractional factorial designs, that use a smart subset of combinations to produce a balanced data set that spans all variations equally, but requires fewer experiments. For example, a full factorial design of five factors requires 32 experiments, while a fractional factorial design can investigate five factors with only eight or 16 experiments. In addition, there are many other designs, like mixture screening designs and various optimization designs.

### Designing a data matrix

When making a regression model, the information contained in a set of predictor variables, $X$, is used to predict variation in a set of responses, $Y$. The most natural application is when the predictors, $X$, actually cause variation in the responses, $Y$. This is called forward causality: using the variables which actually generate variation in the responses as predictors (figure 1).

The forward causality approach to experimental design is the most commonly used, and the one whose properties have been studied most thoroughly. The most classical types of designs stem from that approach, for instance orthogonal designs, which ensure that all $X$ variables vary independently of each other, so that a subsequent regression model can actually be interpreted in terms of causal effects. Mathematically, this strategy ensures optimal properties of the $X$ matrix for building a regression model (calibration).

In reverse causality, the process is inverted: the variables used as predictors $X$ vary because of some amount of

*Table 1. Factors* A, B, *and* C *are varied at two levels. The experimental plan consists of eight experiments* ($=2^3$) *including all combinations of the factors at all levels.*

| *Experiment No.* | *Factor* A | *Factor* B | *Factor* C |
|---|---|---|---|
| 1 | + | + | + |
| 2 | + | + | − |
| 3 | + | − | + |
| 4 | + | − | − |
| 5 | − | + | + |
| 6 | − | + | − |
| 7 | − | − | + |
| 8 | − | − | − |

0142-0453/97 $12·00 © 1997 Taylor & Francis Ltd

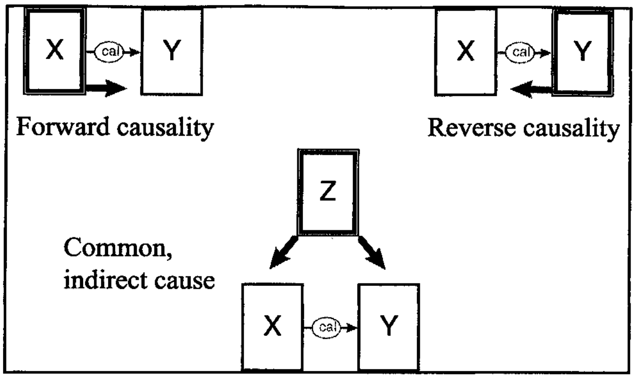

*Figure 1. Three approaches to span variations in data.*

variation in the responses $Y$. Such an approach can be used to span the $Y$-space optimally, thereby ensuring enough variation in $X$ for a successful calibration. Reverse causality, although it seems less natural than forward causality, is the most usual method for calibration of spectroscopic data.

In a third approach, neither $X$ nor $Y$ directly generates variation in the other block of data; an indirect, common cause may induce variations in both $X$ and $Y$. In terms of experimental strategy, the variations are designed in a block of variables $Z$ causing variation in $X$ and $Y$. This third approach ensures that the $X$ and $Y$ values will vary consistently, over a sufficient range. It also ensures some structure in those variations, introduced by the design in the underlying parameters.

One additional advantage of these three causal approaches (besides selecting representative data), is that it is much easier to diagnose any problem with the data (erroneous response values, baseline shifts in the spectra, etc.) than if the samples had been picked at random. The reason is that the structure introduced by the design can easily be visualized, both in the $X$ and $Y$ values, thus any deviation from the expected structure will be spotted immediately.

### *Forward causality—designing* X

This is the traditional approach in experimental design. The experimental conditions ($X$) are varied to produce variations in the measurable responses, for example yield or quality parameters ($Y$) characterizing the sample compositions, in such a way that the $X$ matrix has optimal mathematical properties. This is the basis for orthogonal design. For spectroscopic applications where $X$ is spectra and $Y$ constituents, this approach cannot be applied directly. However, an 'experimental design-inspired' strategy can easily be applied to pick out a balanced set of samples based on the spectral variations.

*Example: investigating important process parameters in a crystallization process*—The producers of the crystalline powder Lacotid used in medicine, wanted to increase the yield (which was only 50%) and make the production more optimal and stable. To achieve this they first needed to find which process parameters have important effects on yield, i.e. establish a cause–effect relationship model.

*Table 2. Experimental variables.*

| *Name* | *Description* | *Units* |
|---|---|---|
| MeOHrest | Rest of methanol MeOH in Lacotid at start | % |
| MeOHprop | Proportion of methanol in methanol + propanol solution | % |
| Feed vel. | Feed velocity of propanol | % |
| Stirring | Stirring speed at addition of propanol | rpm |
| Temp. | Temperature of propanol | °C |
| Cryst. temp. | Lowest crystallization temperature | °C |
| Duration | Duration of crystallization | h |

Seven process parameters were identified as potentially important for the response yield (%) (table 2). Unscrambler software was used to set up an experimental design and to analyse the results. A fractional factorial design was chosen to systematically vary the seven process variables on two levels; $2^{7-3} = 16$ experiments were enough to identify which of the seven design variables have a significant effect on the yield using the classical analysis of effects method (ANOVA), MLT, PLS or another regression method. Once this is known, it is possible to make an optimization experiment based on only the important factors; four replicated centre points were added. Unfortunately it was not possible to perform all the experiments with the stipulated settings of the experimental variables; there were some deviations, which of course were noted. This may cause trouble at analysis of effects. The Unscrambler was used to make a PLS regression model for yield instead. The data in table 3 show the actual settings of the design variables and the measured response values of all the experiments. Note that one experiment has missing response values.

*PLS components*: Two PCs described 82% of the variations of yield. Figure 2 shows sample patterns and variable correlations. The yield is highly negatively correlated

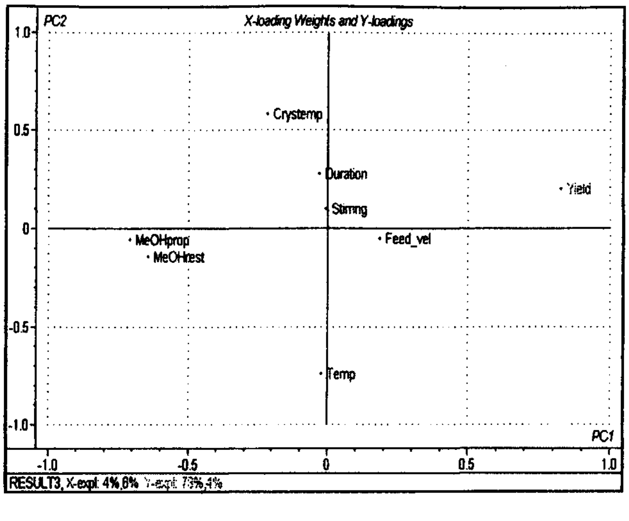

*Figure 2. The score plot for these first two PCs shows the sample patterns. By presenting the samples with their yield value instead of name the best sample is easily identified. In the upper-right corner is the best sample with a yield of 67% (23 June).*

*Table 3. The design variables and the measured response values.*

| | *MeOHrest* | *MeOHprop* | *Fed vel.* | *Stirring* | *Temp.* | *Cryst. temp.* | *Duration* | *Yield* | *OC* |
|---|---|---|---|---|---|---|---|---|---|
| 26 May | 23·80 | 10·00 | 30·00 | 53·00 | 25·00 | 13·00 | 23·50 | 54·00 | 99·50 |
| 25 June | 20·00 | 25·00 | 45·00 | 99·00 | 39·00 | 6·00 | 12·20 | 51·00 | 99·60 |
| 11 June | 18·40 | 40·00 | 11·00 | 153·00 | 24·00 | 10·00 | 5·00 | 55·00 | 98·80 |
| 12 June | 18·40 | 10·00 | 60·00 | 53·00 | 53·00 | 10·00 | 5·00 | 65·00 | 99·40 |
| 29 June | 20·00 | 25·00 | 45·00 | 105·00 | 37·00 | 6·00 | 12·20 | 51·00 | 99·20 |
| 15 June | 18·40 | 40·00 | 30·00 | 53·00 | 46·00 | 10·00 | 20·00 | 55·00 | 99·80 |
| 01 June | 23·80 | 40·00 | 60·00 | 157·00 | 40·00 | 10·00 | 20·70 | 40·00 | 99·60 |
| 16 June | 18·40 | 10·00 | 30·00 | 53·00 | 24·00 | 3·00 | 5·00 | 63·00 | 98·60 |
| 30 June | 20·00 | 25·00 | 45·00 | 102·00 | 38·00 | 6·00 | 12·30 | 51·00 | 99·10 |
| 02 June | 23·80 | 40·00 | 30·00 | 157·00 | 25·00 | 10·00 | 21·30 | 39·00 | 98·90 |
| 17 June | 18·40 | 10·00 | 30·00 | 153·00 | 53·00 | 3·00 | 20·00 | 65·00 | 98·90 |
| 12 June | 23·80 | 10·00 | 30·00 | 157·00 | 50·00 | 10·00 | 5·00 | m | m |
| 03 June | 23·80 | 10·00 | 60·00 | 53·00 | 53·00 | 3·00 | 20·00 | 56·00 | 98·10 |
| 05 June | 23·80 | 40·00 | 30·00 | 60·00 | 49·00 | 3·00 | 5·00 | 37·00 | 99·20 |
| 18 June | 18·40 | 40·00 | 60·00 | 152·00 | 49·00 | 3·00 | 5·00 | 55·00 | 99·60 |
| 09 June | 23·80 | 40·00 | 60·00 | 54·00 | 24·00 | 10·00 | 5·00 | 45·00 | 98·80 |
| 22 June | 18·40 | 40·00 | 60·00 | 53·00 | 25·00 | 3·00 | 20·00 | 61·00 | 100·00 |
| 10 June | 23·80 | 10·00 | 60·00 | 153·00 | 24·00 | 3·00 | 5·00 | 64·00 | 99·00 |
| 23 June | 18·40 | 10·00 | 60·00 | 157·00 | 26·00 | 10·00 | 20·00 | 67·00 | 98·70 |
| 01 July | 20·00 | 25·00 | 45·00 | 105·00 | 37·00 | 6·00 | 12·10 | 52·00 | 98·80 |

m: missing data

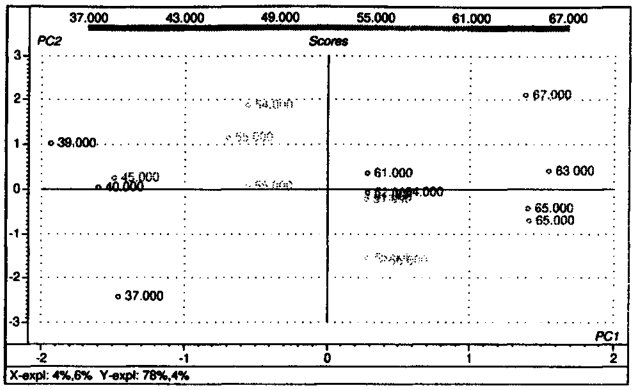

*Figure 3. The loading weights plot shows the variable correlations.*

with MeOHrest and MeOHprop (78% explained variance). The other design variables have less influence on yield. The plot in figure 3 shows that duration of crystallization and stirring speed at addition of propanol has practically no influence at all on yield (only 4% explained variance). The plot explains 78% + 4% = 82% of the total variations of yield, so it gives a pretty clean picture of the relationships expressed as a traditional regression equation:

$$\text{Yield} = b_0 + b_1x_1 + \cdots + b_nx_n$$

The smaller the amount of MeOH in Lacotid at the start of the crystallization, and the smaller the proportion of methanol to propanol, the higher the yield. This is important information when continuing the work to optimize and possibly rebuild the process equipment.

*Next step—optimization*: The highest yield we achieved in the initial screening experiment was 67, at the following process settings:

| | | | |
|---|---|---|---|
| MeOHrest | 18·4 | MeOHprop | 10·0 |
| Feed vel. | 60 | Stirring | 157 |
| Temp. | 26 | Cryst. temp | 10 |
| Duration | 20 | | |

The Unscrambler was used to make a central composite design—a new series of experiments, concentrating on the two (or three) most important factors that were identified in the screening experiment, MeOHrest and MeOHprop. In this case optimization was first tried in the laboratory.

A response surface model was calculated using the Unscrambler. This allows a graphical study of how yield varies with varying levels of the experimental factors; it also allows the optimum settings to be selected either from 3D plot or a contour plot (seen from above) [figure 4]. The optimum yield is approximately 98% at, for example, 14% MeOHrest and 4% MeOHprop. About 90% yield is possible at MeOHrest levels less than 15·5% or more than 17·5%, provided that the MeOHprop ratio is smaller than 4–4·5%. This is due to an interaction effect between MeOHrest and MeOHprop. This interaction effect was not detected at PLS modelling, because the interaction term was not included in the data matrix. The very high yield is probably not achievable in the real process, but the experiments are useful as a guide to how to try and tune the process.

*Reverse causality—designing* Y

This is the most common approach to spectroscopic calibration. The sample compositions are varied in a controlled manner (the constituents *Y*), thus generating variation in the measurable parameters (spectra *X*) which characterize sample composition. This approach ensures optimal spanning of the *Y*-space.

*Example: prediction of alcohol concentrations in a three-compound mixture*—16 samples were produced using a mixture de-

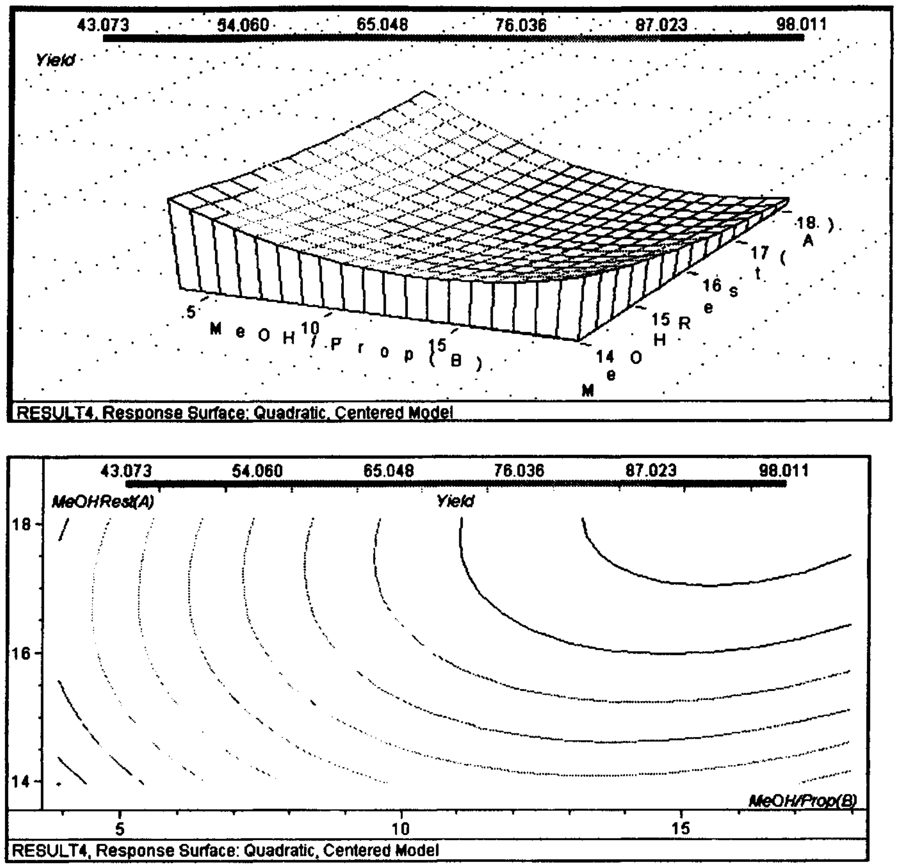

*Figure 4. Response surface of yield.*

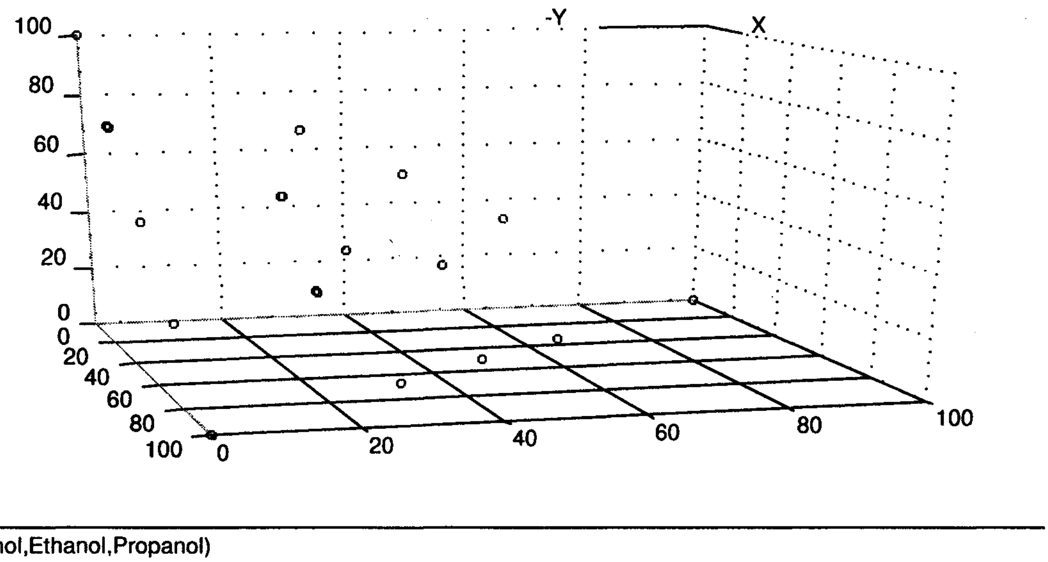

*Figure 5. Triangular mixture design of three components. The corners represent 100% ethanol, 100% propanol and 100% methanol, respectively.*

sign to vary the contents of the three alcohols from 0 to 100%, with the objective being to predict the composition of all possible mixtures [3] of ethanol, propanol and methanol from NIR spectra. A designed *Y* matrix produces a smaller and more uniform error over the whole range of variation. (Figure 5 shows the triangular mixture design.) Ten of the samples were produced again to be used as a separate test set for validation. NIR spectra were recorded with a guide wave instrument from 1100 to 1600 nm at 5 nm intervals.

The Unscrambler [4] software was used to preprocess the spectra with multiplicative scatter correction [3] (MSC) and to make a PLS2 model. By test set validation, a three

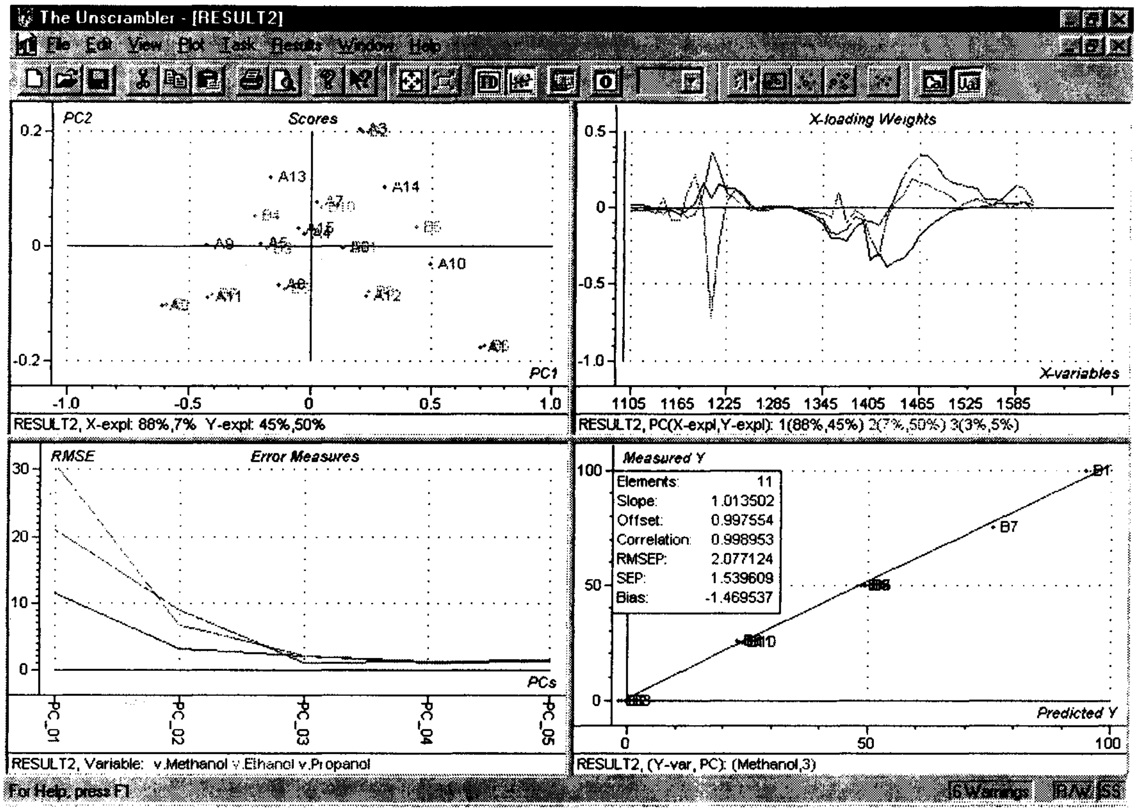

*Figure 6. Regression overview PLS2 of alcohol mixture.*

PLS component (3 PCs) model gave an RMSEP of 2·08 for methanol, 2·0 for ethanol and 1·01 for propanol. Since only two factors were varied independently, only two significant PCs would be expected, but because of interference in the spectra related to non-linearities caused by the mixture, three or four PCs are needed to model all variations in *Y*.

The regression overview in figure 6 shows the sample patterns (score plot), the loading weights spectra for the first three PCs, RMSEP for each of the constituents and predicted vs measured methanol using three PCs.

*Indirect, common causality—designing* Z

*Z* can also be designed—i.e. the variations of experimental conditions or sample composition can be controlled to generate variation in measured parameters for both *X* (for example, spectra) and *Y* (for example response properties). *Z* may be process parameters like pressure, temperature, stirring rate, or ingredients. *Y* may be quality properties. Designing *Z* ensures optimal spanning of both the *X*- and *Y*-space.

*Example: prediction of sensory attributes from chemical properties measured by spectra*—Ellekjær *et al.* [5] produced sausages according to a full factorial design. They varied (*Z*) starch at three levels, salt at three levels, and fat at six levels; in total 54 samples plus a reference sample in triplicate. Spectra (*X*) were recorded by a NIR Technicon instrument 500 from 1100–2500 nm with 4 nm intervals. Sixteen sensory attributes (*Y*) were measured on a scale from 1 to 9 by nine trained assessors, who tasted each sample three times. The objective was to predict sensory measurements from NIR spectra.

The Unscrambler was used to model *Y* from *X* by PLS2, after MSC of the spectra. Twelve PCs describe *ca.* 50% of all the variations in *Y*. Some of the variables were explained to almost 90%, whereas others were only explained to 40–50%. The prediction error RMSEP for some of the sensory variables is given in table 4.

The regression overview in figure 7 shows the distribution of the samples for the two first PCs, the *Y* loadings, the total residual *Y* variance and predicted vs measured for the *Y* variable colour using 12 PCs. The *Y* loading plot for two PCs (30% explained *Y* variance, 97% explained spectral variance) shows the *Y* variable correlations. Smoke odour positively co-varies with smoke flavour and colours, and negatively co-varies with off-flavour and off-odour. Juiciness does not vary much.

There are patterns in the PC1/PC2 score plots in figure 8—the marker names have been replaced by the levels of fat, salt and starch, respectively. The upper-left window shows that variation due to decreasing fat is modelled

*Table 4. RMSEP for some of the sensory variables.*

| *Constituent* | *RMSEP* | *Constituent* | *RMSEP* |
|---|---|---|---|
| Whiteness | 0·29 | Colour | 0·27 |
| Colour intensity | 0·27 | Odour intensity | 0·27 |
| Meat odour | 0·52 | Smoke odour | 0·79 |
| Off-odour | 1·0 | Flavour intensity | 0·6 |
| Spiciness | 0·4 | | |

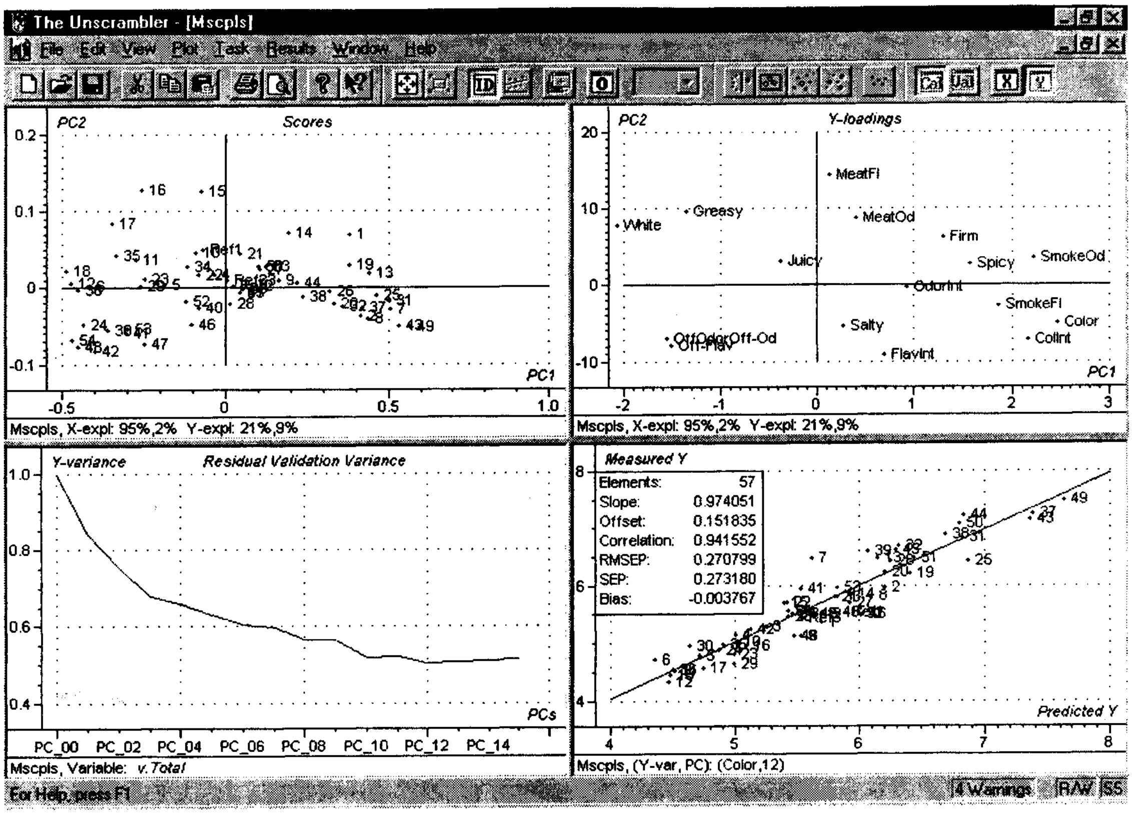

*Figure 7. Regression overview PLS2 sausages.*

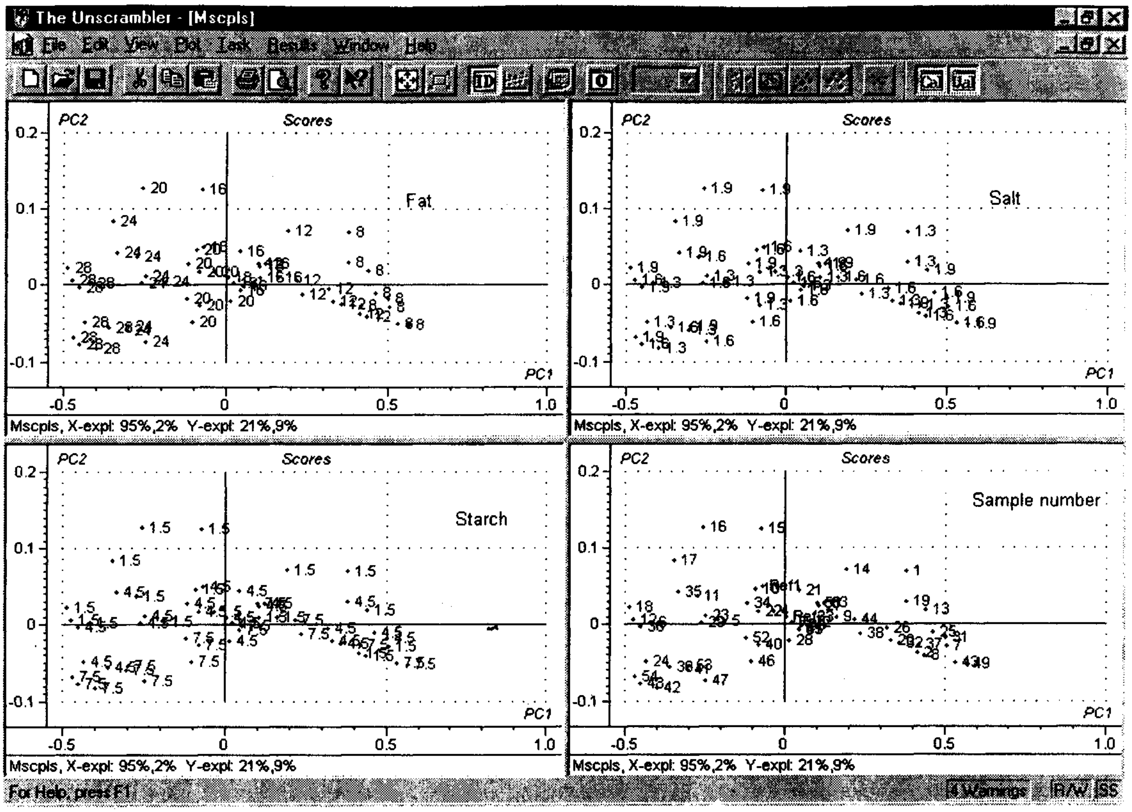

*Figure 8. Score plot of sausages using different markers to show patterns.*

*Table 5. Scores matrix.*

| *Sample name* | *Sample No.* | *Scores PC 01* | *Scores PC 02* | *Scores PC 03* | *Sign pattern* |
|---|---|---|---|---|---|
| M01 | 1 | 0·0292 | 0·0091 | 0·0177 | |
| M02 | 2 | 0·0627 | 0·0188 | 0·0095 | +++ |
| M05 | 3 | 0·0325 | 0·0319 | −0·0258 | |
| L06 | 4 | 0·1250 | −0·0130 | −0·0201 | +−− |
| H11 | 5 | −0·1155 | 0·0016 | −0·0043 | −+− |
| H12 | 6 | −0·1636 | −0·0440 | −0·0161 | |
| L13 | 7 | −0·0033 | −0·0612 | 0·0055 | −−+ |
| L14 | 8 | 0·1258 | −0·0118 | 0·0161 | +−+ |
| L15 | 9 | 0·1504 | −0·0098 | −0·0004 | |
| H17 | 10 | −0·0897 | 0·0501 | 0·0002 | −++ |
| M18 | 11 | 0·0190 | 0·0180 | 0·0150 | |
| H20 | 12 | −0·1318 | −0·0013 | 0·0181 | |
| L21 | 13 | 0·0342 | −0·0532 | 0·0079 | |
| H24 | 14 | −0·1435 | −0·0184 | −0·0079 | |
| H27 | 15 | −0·1418 | −0·0040 | 0·0172 | |
| L29 | 16 | 0·1148 | −0·0169 | 0·0022 | |
| L31 | 17 | 0·1378 | 0·0007 | −0·0037 | |
| H32 | 18 | 0·0141 | 0·0891 | −0·0077 | ++− |
| L35 | 19 | 0·1046 | −0·0232 | −0·0133 | |
| H36 | 20 | −0·0404 | 0·0736 | −0·0066 | |
| L37 | 21 | 0·0539 | −0·0516 | −0·0168 | |
| H38 | 22 | −0·0696 | 0·0709 | −0·0005 | |
| H39 | 23 | −0·2077 | −0·0545 | −0·0040 | −−− |
| L40 | 24 | 0·1030 | −0·0011 | 0·0180 | |

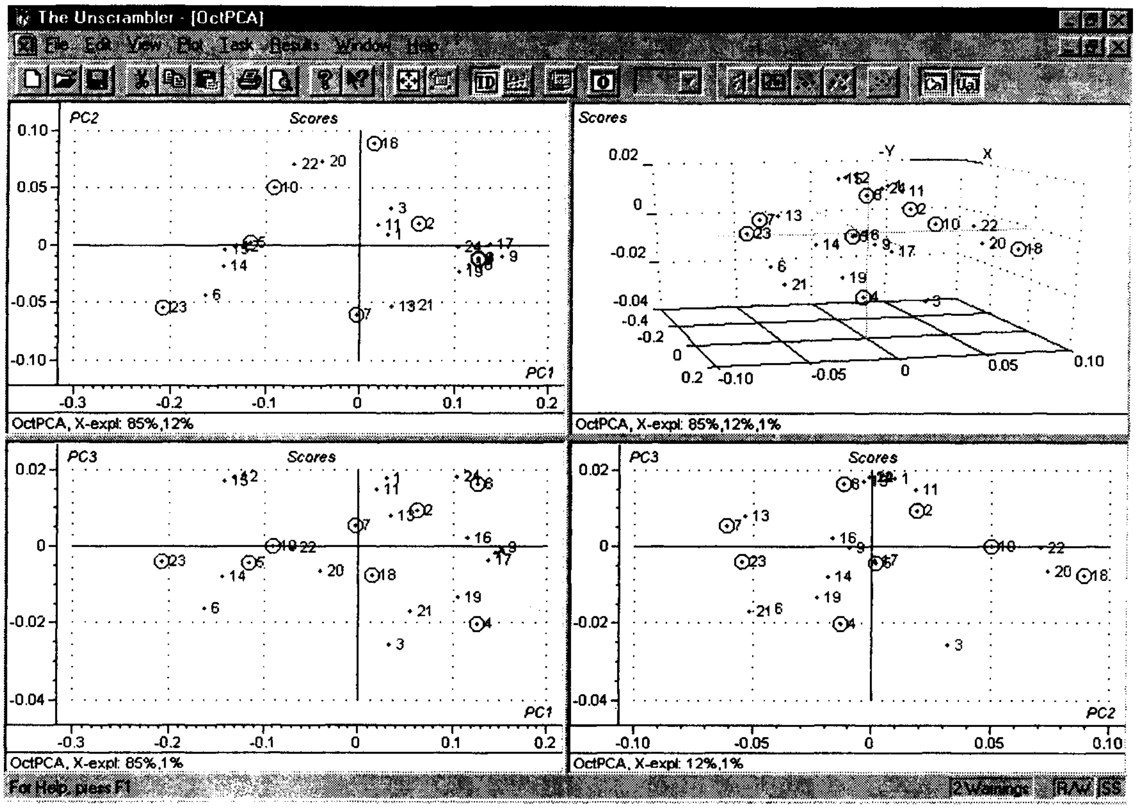

*Figure 9. 3D score plot and its 2D projections. Samples selected by experimental design are circled.*

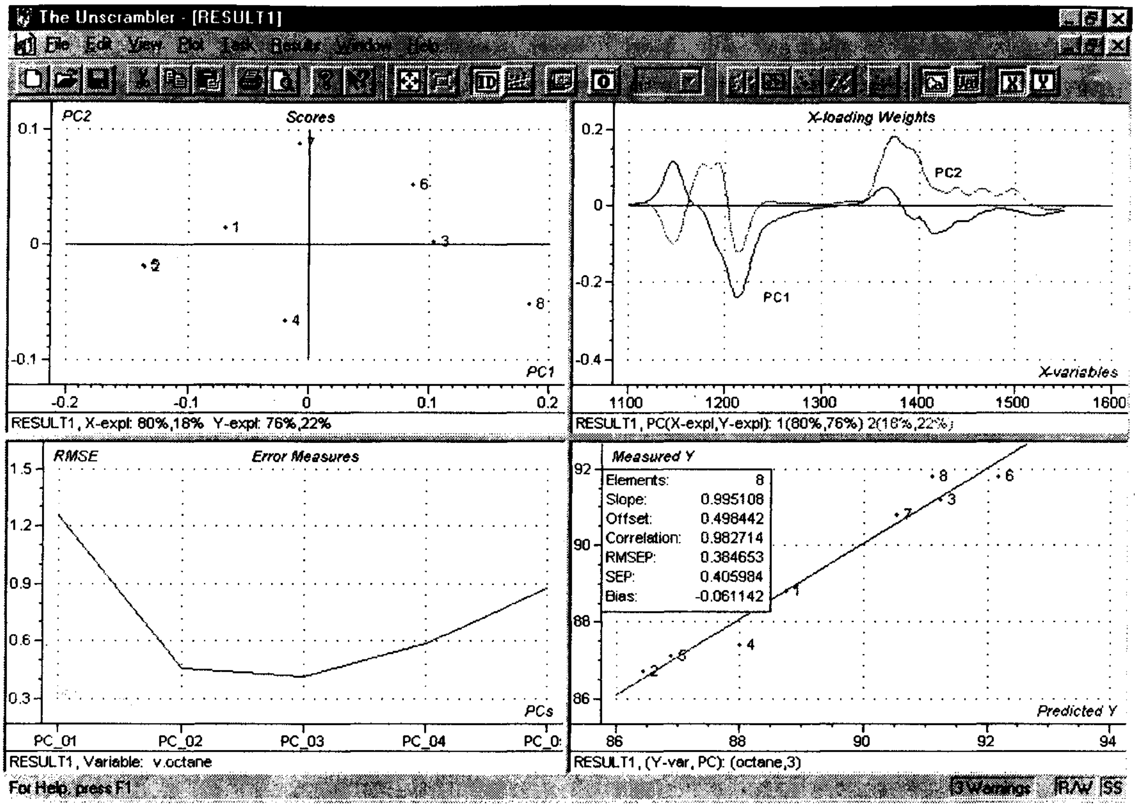

*Figure 10. PLS of selected gasoline samples. RMSEP for octane number is* 0·41 *using 3PCs (leverage correction validation).*

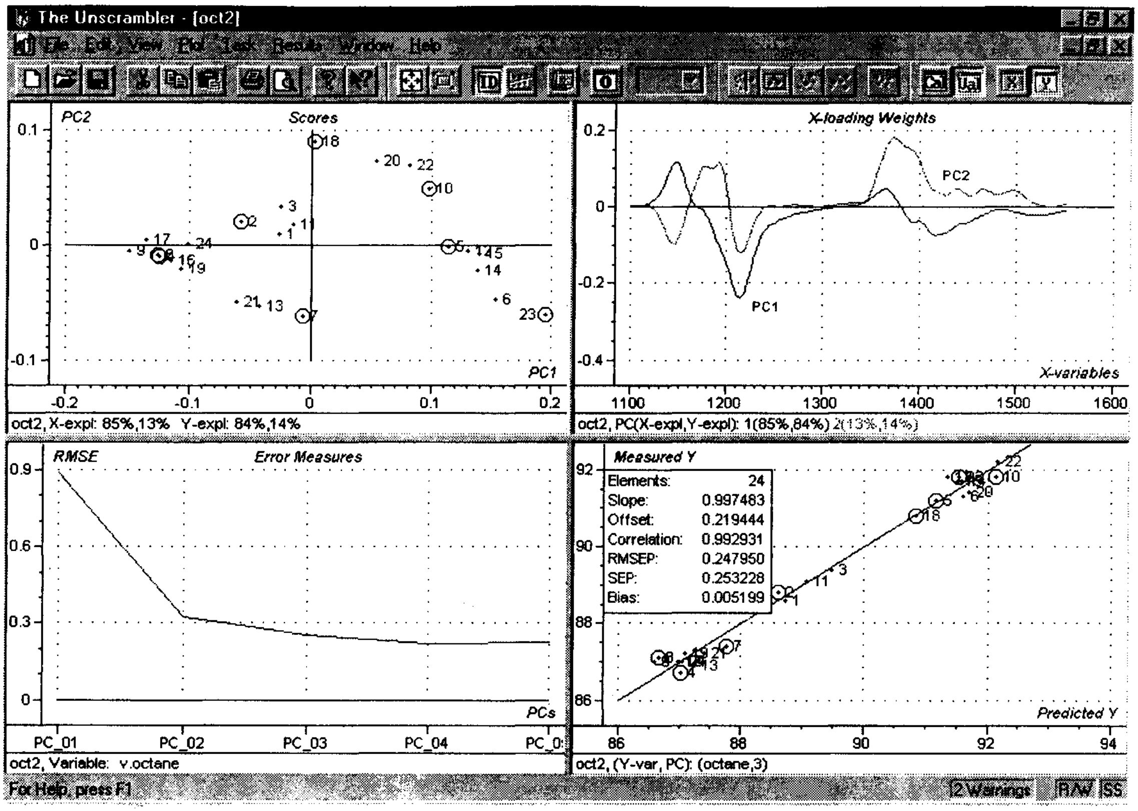

*Figure 11. PLS on 24 gasoline samples, the eight from figure 9 are encircled. RMSEP for octane number (leverage correction) is* 0·25.

along the first PC. The upper right does not show any patterns for salt variation. The lower left shows that the second PC models variations due to decreasing starch.

*Select samples using experimental design*

Forward causality—designing $X$—cannot be done directly on spectra. But it is possible to use an experimental design concept to pick out a balanced set of spectra. A common approach is to make a PCA (principal components analysis) on the spectra, and pick out a set of different samples from the score plots. Average samples are close to the origin in the plot, while more extreme samples are far away from the origin. Samples close to each other are similar, while samples far away from each other are dissimilar.

If, however, the data set needs many principal components (PCs) to be adequately described, it may be difficult to pick out samples evenly from all the PCs. A good approach is to use the systematic pattern of factorial or fractional designs; from the score matrix samples can be picked out with the same pattern of high and low as in factorial design.

*Example: prediction of octane number in gasoline from NIR spectra*—The technique can be illustrated by a set of samples scanned by a guided wave NIR spectrophotometer, using 1100–1550 nm, a 2 nm interval, modelled by the Unscrambler with PCA. Table 3 shows the scores for the first three components, and sign patterns for eight selected samples, corresponding to the patterns of a full factorial design of three factors (table 5).

The 3D score plot in the upper-right window of figure 9 shows the first three PCs. Its projections to PC1/PC2, PC2/PC3 and PC1/PC3, respectively, are shown in the other windows. The samples selected from table 5 are encircled; they are well spread and cover the space in all three PCs well.

Eight samples are usually too few to build a reliable calibration model. However, in this particular example, the eight selected samples gave a PLS model (figure 10) which is not much worse than a PLS model based on all 24 samples (figure 11).

### Conclusions

Depending on the underlying causality structure, there can be three ways to apply experimental design to the selection of adquate data for modelling and calibration. Of course, the same strategy applies for validation data as well. Even if there may be situations where you cannot control the design variables, it may help to think in terms of experimental design when selecting samples, to span all important variations. Factorial or fractional designs with two or more levels, mixture designs or optimization designs like central composite are useful in this context.

### References

1. Carlson, R., *Design and Optimization in Organic Synthesis* (Elsevier, Amsterdam, 1992).

2. Esbesen, K., Midtgaard, T. and Schönkopf, S., *Multivariate Analysis in Practice* (Camo AS, Trondheim 1994).

3. Martens, H. and Næs, T., *Multivariate Calibration* (John Wiley, Chichester, 1989).

4. *The Unscrambler® ver. 6 Software*; and *The Unscrambler User's Guide* (Camo ASA, Ruseløkkveien 6, 0115 OSLO 1986–1996).

5. Ellekjær, M. R., Isaksson, T. and Solheim, R., *Journal of Food Science*, **59** (3) (1994).

